# Tissue transglutaminase (TG2) enables survival of human malignant pleural mesothelioma cells in hypoxia

**DOI:** 10.1038/cddis.2017.30

**Published:** 2017-02-02

**Authors:** Sara Zonca, Giulia Pinton, Zhuo Wang, Maria Felicia Soluri, Daniela Tavian, Martin Griffin, Daniele Sblattero, Laura Moro

**Affiliations:** 1 Department of Pharmaceutical Sciences, University of Piemonte Orientale, Novara, Italy; 2 School of Life and Health Sciences, Aston University, Birmingham, UK; 3 Department of Health Sciences, University of Piemonte Orientale, Novara, Italy; 4 Laboratory of Cellular Biochemistry and Molecular Biology, CRIBENS, Catholic University of the Sacred Heart, Milan, Italy; 5 Department of Life Sciences, University of Trieste, Trieste, Italy

## Abstract

Malignant pleural mesothelioma (MPM) is an aggressive tumor linked to environmental/occupational exposure to asbestos, characterized by the presence of significant areas of hypoxia. In this study, we firstly explored the expression and the role of transglutaminase 2 (TG2) in MPM cell adaptation to hypoxia. We demonstrated that cells derived from biphasic MPM express the full-length TG2 variant at higher levels than cells derived from epithelioid MPM and normal mesothelium. We observed a significant induction of TG2 expression and activity when cells from biphasic MPM were grown as a monolayer in chronic hypoxia or packed in spheroids, where the presence of a hypoxic core was demonstrated. We described that the hypoxic induction of TG2 was HIF-2 dependent. Importantly, *TGM2-v1* silencing caused a marked and significant reduction of MPM cell viability in hypoxic conditions when compared with normoxia. Notably, a TG2-selective irreversible inhibitor that reacts with the intracellular active form of TG2, but not a non-cell-permeable inhibitor, significantly compromised cell viability in MPM spheroids. Understanding the expression and function of TG2 in the adaptation to the hypoxic environment may provide useful information for novel promising therapeutic options for MPM treatment.

Malignant pleural mesothelioma (MPM) is an aggressive tumor linked to exposure to asbestos fibers, arising from mesothelial surfaces of the pleural cavity.^1, 2^ Asbestos-related diseases develop slowly and are often diagnosed in their later stages, preventing many patients from achieving treatment at the most crucial point of disease progression.^3^

Very few patients are eligible for curative surgical resection, and radiation therapy has also demonstrated poor results, rendering chemotherapy the treatment of choice.^4^

Standard chemotherapeutic regimens in MPM are the combination of pemetrexed and cisplatin or carboplatin. Nevertheless, MPM is often refractory to chemotherapeutic agents, and treatment intent is mostly palliative.^5, 6, 7^

It is therefore necessary to acquire new insights into the pathobiology of this disease to improve diagnosis and provide targets for more effective therapeutic strategies.

Transglutaminase 2 (TG2) is the first identified member of a family of structurally and functionally related proteins widely distributed in different tissues and cell types.^8^

TG2 catalyses the Ca^2+^-dependent post-translational modifications of proteins by introducing covalent bonds between free primary amine groups and peptide bound glutamine residues. It also displays GTPase, ATPase, protein kinase and protein disulfide-isomerase activity.^9, 10, 11, 12^ In addition, TG2 mediates the interaction of integrins and syndecans^13^ with fibronectin and crosslinks proteins of the extracellular matrix, when it is externalized from cells.^14^ TG2 overexpression has been observed in many tumors, including pancreatic, breast, colon, ovarian, non-small cell lung cancers, glioblastoma and melanoma,^15, 16, 17, 18, 19, 20^ whereas no data are present in literature concerning its expression and function in MPM.

Increased expression of TG2 in cancer cells has been linked to drug resistance, metastasis and poor patient survival.^21^ Under physiological conditions, in the intracellular environment, there is low Ca^2+^ and a high GTP/GDP ratio and TG2 is in an inactive form.^22^ Under stress conditions, TG2 can be regulated by the activation of several signaling pathways or epigenetic mechanisms.^23^ It has been demonstrated that induced TG2 expression confers a growth advantage to cancer cells to survive in hypoxic conditions.^24^ Hypoxia can promote tumor progression and resistance to the effects of chemotherapy and radiation; however, it is well known that it can also inhibit tumor cell proliferation.^25, 26^ Cellular adaptation to micro-environmental hypoxia lets tumor cells undergo some changes, including metabolic transformation. These adaptive responses are mainly driven by HIFs, the hypoxia-inducible transcription factors. HIFs are heterodimers comprising one of three major oxygen labile HIF-*α* subunits (HIF-1*α*, HIF-2*α* or HIF-3*α*) and a constitutive HIF-1*β* subunit (also known as aryl hydrocarbon receptor nuclear translocator or ARNT), which together form the HIF-1, HIF-2 and HIF-3 transcriptional complexes, respectively.^27^ Intriguingly, it appears that in some cell lines, HIF-1 drives the initial response to hypoxia while during chronic hypoxic exposure it is HIF-2 that has the major role in driving the hypoxic response.^28, 29, 30^ Recent studies confirmed that MPM is a tumor with significant hypoxic areas in the dominant tumor masses.^31^

TG2 has been described as undertaking important roles in tumorigenesis, tumor differentiation and invasion, however a role for TG2 in MPM has not been reported yet. In this study, we measured TG2 expression and enzymatic activity in MPM cell lines in the adaptation to the hypoxic environment.

## Results

### Constitutively spliced full-length TG2 is highly expressed in MPM cell lines

The expression levels of the constitutively spliced full-length *TGM2-v1* and of four *TGM2* variants (*TGM2-v2, v3, v4A, v4B*), generated by alternative splicing,^32^ were evaluated by real-time PCR in mesothelium and MPM derived cell lines.

**Figure 1:**
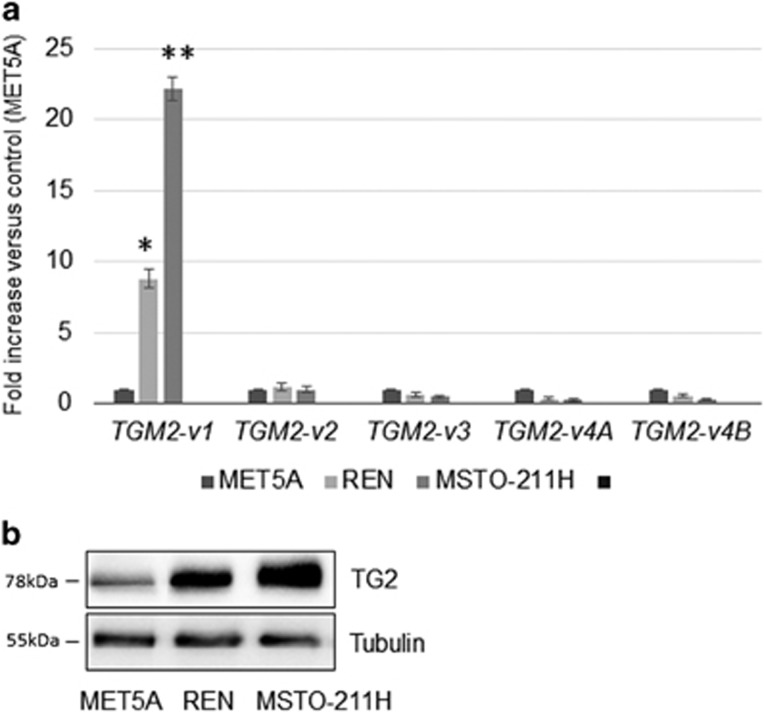
TG2 expression in mesothelium and mesothelioma derived cell lines. (**a**) Real-time PCR analysis of *TGM2-v1*, *TGM2-v2, TGM2-v3, TGM2-v4A* and *TGM2-v4B* mRNA in MET5A, REN and MSTO-211H cells. 18S rRNA was used as housekeeping gene. Each bar represents mean of three independent experiments±S.D. *P≤0.05, **P≤0.01. (**b**) Representative western blot analysis of TG2 expression in MET5A, REN and MSTO-211H cells. Tubulin was used as loading control

We demonstrated, by real-time PCR experiments, that the constitutively spliced *TGM2-v1* transcripts were increased in REN and MSTO-211H cells, derived from epithelioid and biphasic MPMs (9-fold and 22-fold increase, respectively) if compared with mesothelial MET5A cells (Figure 1a). Notably, the increased expression of the full-length TG2 in tumor cells was confirmed at protein level, by western blot analysis (Figure 1b).

### TG2 expression and enzymatic activity increase and prevent MSTO-211H cell death in hypoxia

To investigate whether TG2 has an important role in MPM derived cells in normoxic or hypoxic environments, we transiently transfected TG2 siRNA into MSTO-211H cells, which express the highest levels of the protein.

**Figure 2:**
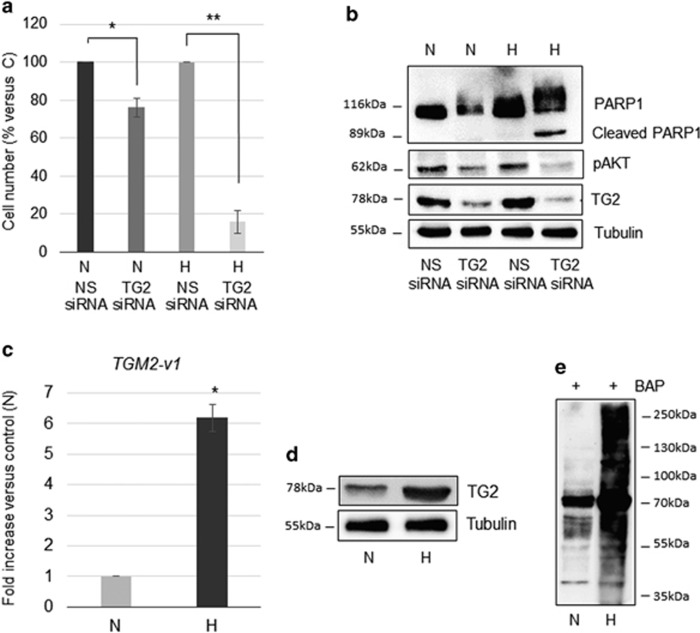
TG2 expression and enzymatic activity increase and prevent MSTO-211H cell death in hypoxia. (**a**) Bar graph shows the percentage of viable MSTO-211H cells transfected with siRNA specific for *TGM2-v1* (TG2 siRNA) versus non-specific siRNA (NS siRNA), cultured 48 h in normoxia (N) or hypoxia (H). Each bar represents mean of three independent experiments±S.D. *P≤0.05, **P≤0.01. (**b**) Representative western blot analysis of PARP1, cleaved PARP1, pAKT and TG2 expression in MSTO-211H cells transfected with non-specific siRNA (NS siRNA) or siRNA specific for *TGM2-v1* (TG2 siRNA) and cultured 48 h in normoxia (N) or hypoxia (H). Tubulin was used as loading control. (**c**) Real-time PCR analysis of *TGM2-v1* mRNA in MSTO-211H cells cultured 48 h in normoxia (N) or hypoxia (H). 18S rRNA was used as housekeeping gene. Each bar represents mean of three independent experiments±S.D. *P≤0.05. (**d**) Representative western blot analysis of TG2 expression in MSTO-211H cells cultured 48 h in normoxia (N) or hypoxia (H). Tubulin was used as loading control. (**e**) Representative western blot of the TG2-catalyzed incorporation of 5-(biotinamido) pentylamine (BAP) into proteins in MSTO-211H cells cultured 48 h in normoxia (N) or hypoxia (H)

As shown in Figure 2a, *TGM2-v1* silencing significantly compromised cell viability in hypoxia, while exerting only a moderate effect in normoxic condition. By western blot analysis, shown in Figure 2b, we confirmed silencing and evidenced an increase in PARP1 cleavage along with reduced AKT phosphorylation in cells silenced for *TGM2-v1*, when grown in hypoxia. On the contrary, we observed increased proliferation in MSTO-211H cells transfected with an expression vector coding for (GFP)-TG2, cultured in hypoxia (Supplementary Figure S1A and B). We next measured the expression of *TGM2-v1* transcript in MSTO211-H cells cultured in normoxic or hypoxic conditions. TG2 was found to be significantly upregulated at mRNA and protein level after 48 h of cell culture in hypoxia, when compared with normoxia (Figure 2c and d). Furthermore, we assessed the transamidating activity in both conditions by incorporation of 5-(biotinamido)pentylamine (BAP) into proteins. As shown in Figure 2e, transamidating activity was significantly enhanced in MSTO-211H cells grown 48 h in hypoxic conditions compared with cells grown in normal atmospheric oxygen concentration. TG2 induced expression and its role in enabling cell survival in hypoxia were observed in MET5A and REN cells too (Supplementary Figure S2A and B).

### TG2 expression depends on HIF-2 and is induced in the hypoxic core of MSTO-211H spheroids

**Figure 3:**
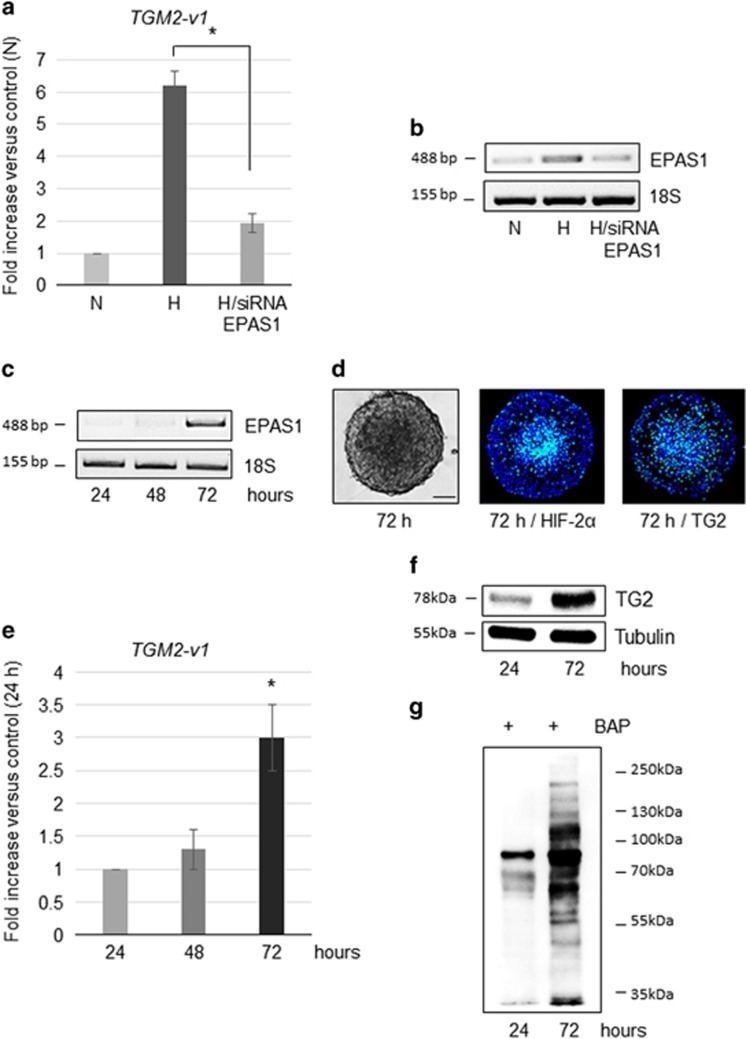
TG2 expression depends on HIF-2 and is induced in the hypoxic core of MSTO-211H spheroids. (**a**) Real-time PCR analysis of *TG2M-v1* mRNA in MSTO-211H cells transfected with non-specific siRNA or specific for *EPAS1* (siRNA *EPAS1*) and cultured 48 h in normoxia (N) or hypoxia (H). 18S rRNA was used as housekeeping gene. Each bar represents mean of three independent experiments±S.D. *P≤0.05. (**b**) Representative quantitative RT-PCR analysis of *EPAS1* in MSTO-211H cells transfected with non-specific control siRNA or *EPAS1* specific siRNA (siRNA EPAS1) and cultured 48 h in normoxia (N) or hypoxia (H). 18S rRNA was used as housekeeping gene. (**c**) Representative quantitative RT-PCR analysis of *EPAS1* in MSTO-211H cells grown for 24, 48 or 72 h as spheroids (pools of 5). 18S rRNA was used as housekeeping gene. (**d**) Phase contrast images and immunofluorescence analysis of HIF-2*α* and TG2 spatial distribution in MSTO-211H spheroids at 72 h, evidenced by FITC-conjugated secondary Abs (× 200 magnification). Bar equals 100 *μ*M. (**e**) Real-time PCR analysis of *TG2M-v1* mRNA in MSTO-211H cells grown 24, 48 or 72 h as spheroids. 18S rRNA was used as housekeeping gene. Each bar represents mean of three independent experiments±S.D. *P≤0.05. (**f**) Representative western blot analysis of TG2 expression in MSTO-211H cells grown 24 or 72 h as spheroids. Tubulin was used as loading control. (**g**) Representative western blot of the TG2-catalyzed incorporation of 5-(biotinamido) pentylamine (BAP) into proteins in MSTO-211H cells grown 24 or 72 h as spheroids

As observed by us and reported in literature,^30^ HIF-2 has the major role in driving response to chronic hypoxia. We tested the effect of *EPAS1* silencing on *TGM2-v1* expression in MSTO-211H cells cultured 48 h in hypoxia. Data, shown in Figure 3a, confirm that the induction of *TG2M-v1* expression was dependent on HIF-2*α*. The *EPAS1* induction in hypoxia and its silencing were confirmed by semi-quantitative RT-PCR (Figure 3b). Next, we cultured MSTO-211H cells, for 24, 48 or 72 h, as multicellular 3D spheroids. *EPAS1* expression and HIF-2*α* localization in the hypoxic core of spheroids at 72 h were confirmed by RT-PCR and immunofluorescence staining with specific antibodies (Figure 3c and d). Parallel induction of *TGM2-v1* at 72 h in spheroids was assessed by real-time PCR (Figure 3e). As shown in Figure 3f and g, western blot analyses confirmed that both TG2 expression and transamidating activity increased in spheroids at 72 h compared to spheroids at 24 h. TG2 localization in spheroids at 72 h was analyzed by immunofluorescence staining with specific antibodies (Figure 3d).

### TG2 silencing or intracellular inhibition compromises the MSTO-211H cell viability in multicellular spheroids

**Figure 4:**
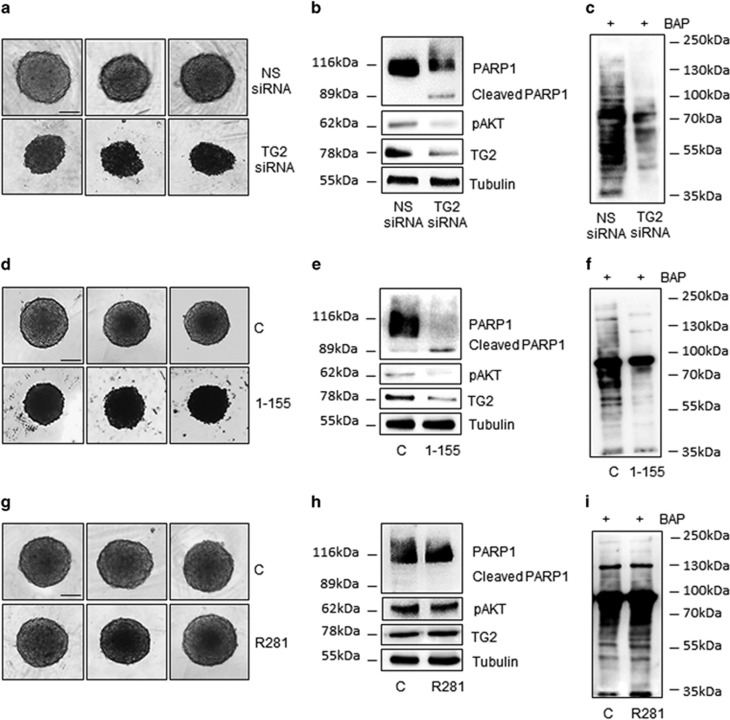
TG2 silencing or intracellular inhibition compromises the MSTO-211H cell viability in multicellular spheroids. (**a**) Phase contrast images (× 200 magnification) of MSTO-211H cells transfected with non-specific siRNA (NS siRNA) or specific for *TGM2-v1* (TG2 siRNA) and grown 72 h as spheroids. Bar equals 100 *μ*M. (**b**) Representative western blot analysis of PARP1, cleaved PARP1, pAKT and TG2 expression in MSTO-211H cells transfected with non-specific siRNA (NS siRNA) or specific for *TGM2-v1* (TG2 siRNA) and grown 72 h as spheroids. Tubulin was used as loading control. (**c**) Representative western blot of the TG-catalyzed incorporation of 5-(biotinamido) pentylamine (BAP) into proteins in MSTO-211H transfected with non-specific siRNA (NS siRNA) or specific for *TGM2-v1* (TG2 siRNA) and grown 72 h as spheroids. (**d**) Phase contrast images (× 200 magnification) of MSTO-211H cells grown 72 h as spheroids±25 *μ*M 1–155. Bar equals 100 *μ*M. (**e**) Representative western blot analysis of PARP1, cleaved PARP1, pAKT and TG2 expression in MSTO-211H cells grown 72 h as spheroids±25 *μ*M 1–155. Tubulin was used as loading control. (**f**) Representative western blot of the TG2-catalyzed incorporation of BAP into proteins in MSTO-211H cells grown 72 h as spheroids±25 *μ*M 1–155. (**g**) Phase contrast images (× 200 magnification) of MSTO-211H cells grown 72 h as spheroids±25 *μ*M R281. Bar equals 100 *μ*M. (**h**) Representative western blot analysis of PARP1, cleaved PARP1, pAKT and TG2 expression in MSTO-211H cells grown 72 h as spheroids±25 *μ*M R281. Tubulin was used as loading control. (**i**) Representative western blot of the TG2-catalyzed incorporation of BAP into proteins in MSTO-211H cells grown 72 h as spheroids±25 *μ*M R281

We next examined the effect of *TGM2-v1* silencing in MSTO-211H spheroids. As shown in Figure 4a, *TGM2-v1* silencing significantly compromised cell viability in spheroids that appeared reduced in size and dark in light microscopy. Furthermore, *TGM2-v1* silencing induced PARP1 cleavage and reduced AKT phosphorylation, after 72 h (Figure 4b). Remarkably, these results were reproduced by the use of 25 *μ*M 1–155, a cell-permeable selective TG2 inhibitor (Figure 4d and e), whereas no evident effects were observed after incubation of spheroids with 25 *μ*M of the non-cell-permeable TG2 inhibitor R281 (Figure 4g and h). Furthermore, we assessed the transamidating activity in all conditions, by incorporation of the competitive amine substrate BAP into proteins. As shown in Figure 4c and f, transamidating activity was significantly reduced in MSTO-211H spheroids silenced for TG2 expression or treated with 1–155 inhibitor, whereas it was not compromised by incubation with the R281 inhibitor at the same concentration (Figure 4i). Transamidating activity was however abolished when spheroids were incubated with a ten times (i.e., 250 *μ*M) higher dose of R281 (Supplementary Figure S3A), even though neither spheroids viability nor PARP cleavage or AKT phosphorylation resulted compromised (Supplementary Figure S3B and C).

## Discussion

In this study, we firstly describe that tumor cell lines derived from MPM patients express higher basal levels of TG2 compared with normal mesothelial cells. Furthermore, we show that in cells derived from biphasic MPM, the levels of expressed TG2 and transamidating activity significantly increase in response to chronic hypoxia.

Hypoxia is a common feature of solid tumors and is associated with disease progression as well as resistance to radiotherapy and chemotherapy.^33, 34^ However, the presence of hypoxic areas is a feature of cancer that can be exploited for designing rational therapeutic approaches.^35^

Recently published data on hypoxia imaging provided evidence that human MPMs are characterized by the presence of significant areas of hypoxia.^31^ Differently from other tumors in which the hypoxic condition induces epithelial-mesenchymal transition and invasion,^36, 37^ we have recently described that hypoxia causes the switch from spindle to epithelioid phenotype, along with reduced growth rate, in cells derived from biphasic MPM.^38^

TG2 has crucial roles in a number of both physiological and pathological events, which involve modifying its substrate proteins.^39^ Numerous studies that investigated the putative role of TG2 in cancers described the dual role of the protein acting either as a facilitator or attenuator of cell proliferation.^40^ The evidence that TG2 is activated by reactive oxygen species implies TG2 acts as a stress responder that may confer a growth advantage to cancer cells to survive in micro-environmental hypoxia.^41^

To investigate the effect of TG2 on MPM cells under hypoxia, we established MPM hypoxic cell models *in vitro,* by placing cells in an environmentally controlled chamber in which oxygen levels in the gas phase were maintained at 1%. Furthermore, we used multicellular spheroid as a 3D model that accurately reproduces the oxygen and nutrient distribution of avascular tumor masses and measured the expression and activity of TG2 at different time points. The results indicate that, together with the enhanced expression of TG2 mRNA and protein, transamidating activity also increased under prolonged hypoxia.

The response to hypoxia is primarily mediated by the family of HIF transcription factors, regulated by the oxygen-sensing HIF hydroxylases.^42^ HIF-2*α* was initially identified as the endothelial PAS domain protein, but it is also expressed in many other tissues and exerts a widespread role in driving the response during chronic hypoxia exposure.^30^

Our data demonstrate that TG2 expression is under the control of HIF-2 and is crucial for the hypoxia response of MPM cells. In fact, gene silencing revealed only a minor role for TG2 in normoxia, whereas under hypoxic conditions cell viability was severely compromised.

The reduction in AKT phosphorylation, along with the increase in PARP1 cleavage, observed in both *TGM2-v1*-silenced cells and 1–155 treated cells under hypoxia, is suggestive of induced apoptosis. However, the regulatory mechanisms of TG2 in cell death require additional studies. Interestingly, downregulation of TG2 expression also occurred in the 1–155 treated cells, suggesting that TG2 activity can regulate its own expression in the MPM cells by a positive feedback mechanism. Such mechanisms involving either the activation of TGF-*β* or NF-kB by TG2, which in turn regulate TG2 expression have been reported by others and recently reviewed by Huang *et al.*^41^

Furthermore, the significantly impaired transamidating activity, observed in MSTO-211H spheroids silenced for *TGM2-v1* expression, is suggestive that the observed enzymatic activity is mainly mediated by this TG isoform.

Notably, the treatment with 1–155, a TG2-selective irreversible inhibitor that can react with the intracellular active form of the enzyme, significantly compromised MPM cell viability when grown as spheroids. Moreover, it has been described that this small-molecule inhibitor, which can react with TG2 inside the cell, holding the enzyme in its open conformation, prevents its export and translocation into the ECM, events which are paralleled by a reduction in the deposition of the FN matrix.^43, 44^

Further studies, using high doses of the non-cell-permeable R281 inhibitor, confirmed that the extracellular TG2-transamidating activity does not have a role in preventing MPM cell apoptosis under hypoxic stress. As recently suggested,^45, 46^ our data indicate that TG2 could act as a signaling/scaffold protein or mediate some kind of activity that requires the closed GTP bound conformation. However, at the moment we cannot fully rule out a role for intracellular TG2-transamidating activity as it was monitored by detecting BAP incorporation and we have no direct evidence that this agent is cell permeating in our cell model.

In summary, our findings demonstrate that the expression and function of TG2 has an important role in the adaptation of MPM cell to hypoxia and therefore inhibition of TG2 may provide a novel therapeutic avenue for the treatment of this disease.

## Materials and Methods

### Reagents and antibodies

The polyclonal antibody specific for pAKT and the monoclonal antibodies specific for PARP1, HIF-2*α* and *α*-tubulin were purchased from Santa Cruz Biotechnology (Santa Cruz CA, USA). Anti-mouse and anti-rabbit IgG peroxidase or fluorescein isothiocyanate (FITC)-conjugated antibodies and chemical reagents were from Sigma-Aldrich (St Louis, MO, USA). Enhanced chemiluminescence (ECL), nitrocellulose membranes and protein assay kit were from Bio-Rad (Hercules, CA, USA). The monoclonal antibody specific for TG2 (TG100), culture media, sera, antibiotics and LipofectAMINE transfection reagent were from Thermo Fisher (Waltham, MA, USA). The highly selective TG2 inhibitors, 1–155 (cell-permeable) and R281 (non-cell-permeable), were designed and synthesized as previously documented.^47, 48^

### Cell cultures and transfection

The mesothelium derived MET5A and the biphasic MPM MSTO-211H cell lines were obtained from the Istituto Scientifico Tumori (IST) Cell-bank, Genoa, Italy; the epithelioid MPM REN cell line was isolated, characterized and kindly provided by Dr Albelda SM (University of Pennsylvania, Philadelphia; PA, USA). Cells were grown in standard conditions in RPMI medium supplemented with 10% FBS, 100 *μ*g/ml streptomycin and 10 *μ*g/ml penicillin at 37 °C in a humidified environment containing 5% CO2. Cell cultures under hypoxic conditions were performed in 1% O2 gas mixture using a modulator incubator chamber. Mycoplasma infection was excluded by the use of Mycoplasma PlusTM PCR Primer Set kit from Stratagene (La Jolla, CA, USA). Cells grown to 80% confluence in tissue culture dishes were transiently transfected with negative control or specific siRNAs (1027416, 1027416) from Qiagen (Hilden, Germany) using LipofectAMINE reagent as described by the manufacturer. Following treatments, cells were trypsinized and stained with Trypan blue. The number of cells considered viable (unstained cells) was counted in a Bürker haemocytometer within 5 min after staining.

### Multicellular spheroids

Multicellular spheroids were generated in non-adsorbent round-bottomed 96-well plates, as previously described.^49^ The 96-well plates were coated with a 1 : 24 dilution of polyHEMA (120 mg/ml) in 95% ethanol and dried at 37 °C for 24 h. Before use, plates were sterilized by UV light for 30 min. For generation of multicellular spheroids, 5 × 10^3^ cells were added into each well of polyHEMA-coated 96-well plate and placed in a 37 °C humidified incubator with 5% CO_2_. Every 24 h, 50% of supernatant was replaced with fresh medium±1–155 or R281 TG2 inhibitors (at final concentration of 25 *μ*M).

### Immunofluorescence staining

Cell spheroids were fixed in 4% paraformaldehyde, permeabilized with 0.5% Triton X-100 in PBS and blocked in 3% BSA/PBS 10% FBS. The primary antibodies (mouse anti-HIF-2*α* or mouse anti TG2; 1 : 100) were incubated for 2 h at 4 °C. The fluorescent secondary antibody (rabbit anti-mouse IgG antibody conjugated with FITC; 1 : 100) was added and incubated for 1 h at 4 °C. The cell nuclei were counter-stained with 4′,6-diamidino-2-phenylindole. Fluorescent images were captured using a Leica MB5000B microscope equipped with a DFC480 R2 digital camera and a Leica Application Suite (LAS) software.

### Cell lysis and immunoblot

Cells were extracted with 1% NP-40 lysis buffer (1% NP-40, 150 mM NaCl, 50 mM Tris-HCl pH 8.5 10 mM EDTA, 10 mM NaF, 10 mM Na_4_P_2_O_7_, 0.4 mM Na_3_VO_4_) with freshly added protease inhibitors (10 *μ*g/ml leupeptin, 4 *μ*g/ml pepstatin and 0.1 Unit/ml aprotinin). Lysates were centrifuged at 13 000 × *g* for 10 minutes at 4 °C and the supernatants were collected and assayed for protein concentration with the Bradford assay method (Bio-Rad).

Proteins were separated by SDS-PAGE under reducing conditions. Following SDS-PAGE, proteins were transferred to nitrocellulose, reacted with specific antibodies and then detected with peroxidase-conjugate secondary antibodies and chemioluminescent ECL reagent. Digital images were taken with the Bio-Rad ChemiDoc Touch Imaging System and quantified using Bio-Rad Image Lab 5.2.1.

### Measurement of TG2 activity in intact cells

TG2 activity was determined by pre-incubating cells, cultured in 2D or as spheroids, with 1 mM 5-biotinamido-pentylamine, a competitive amine substrate of TG2, for 2 or 24 h at 37 °C. Cells were then washed in PBS and lysed with 1% NP-40 lysis buffer and protein content in the supernatant was quantified. Western blot analysis was performed on the cell lysate with peroxidase-conjugate streptavidin and ECL reagent.

### RNA isolation and Real-time PCR

Total RNA was extracted using the guanidinium thiocyanate method. Starting from equal amounts of RNA, cDNA used as template for amplification in the real-time PCR (5 *μ*g), was synthesized by the reverse transcription reaction using RevertAid Minus First Strand cDNA Synthesis Kit from Fermentas-Thermo Scientific (Burlington, ON, Canada), using random hexamers as primers, according to the manufacturer's instructions. Twenty nanogram of cDNA were used to perform RT-PCR amplification of *EPAS1* mRNA. The primers sequences for *EPAS1* were: Fw 5′-GGGGATCAGCGCACAGAGTT-3′ and Rev 5′-TGGGCTGACGACAGGCTGTA-3′ 18S RNA was simultaneously amplified using the primers: Fw 5′-AAACGGCTACCACATCCAAG-3′ and Rev 5′-CCTCCAATGGATCCTCGTTA-3′. The real-time reverse transcription-PCR was performed using the double-stranded DNA-binding dye SYBR Green PCR Master Mix (Fermentas-Thermo Scientific) on an ABI GeneAmp 7000 Sequence Detection System machine, as described by the manufacturer. The instrument, for each gene tested, obtained graphical cycle threshold (Ct) values automatically. Triplicate reactions were performed for each marker and the melting curves were constructed using Dissociation Curves Software (Applied Biosystems, Foster City, CA, USA), to ensure that only a single product was amplified. We used the isoform specific oligonucleotide primers pairs designed by Phatak VM *et al.*^32^ to selectively amplify *TGM2* transcripts.

### Statistical analysis

Statistical evaluation of the differential analysis was performed by one-way ANOVA and Student's *t*-test.
