# Supplementary figures and images for: Tissue transglutaminase (TG2) enables survival of human malignant pleural mesothelioma cells in hypoxia
Source: Cell Death Dis. 2017 Feb 2;8(2):e2592–. doi: 10.1038/cddis.2017.30 (PMC5386478; doi:10.1038/cddis.2017.30)

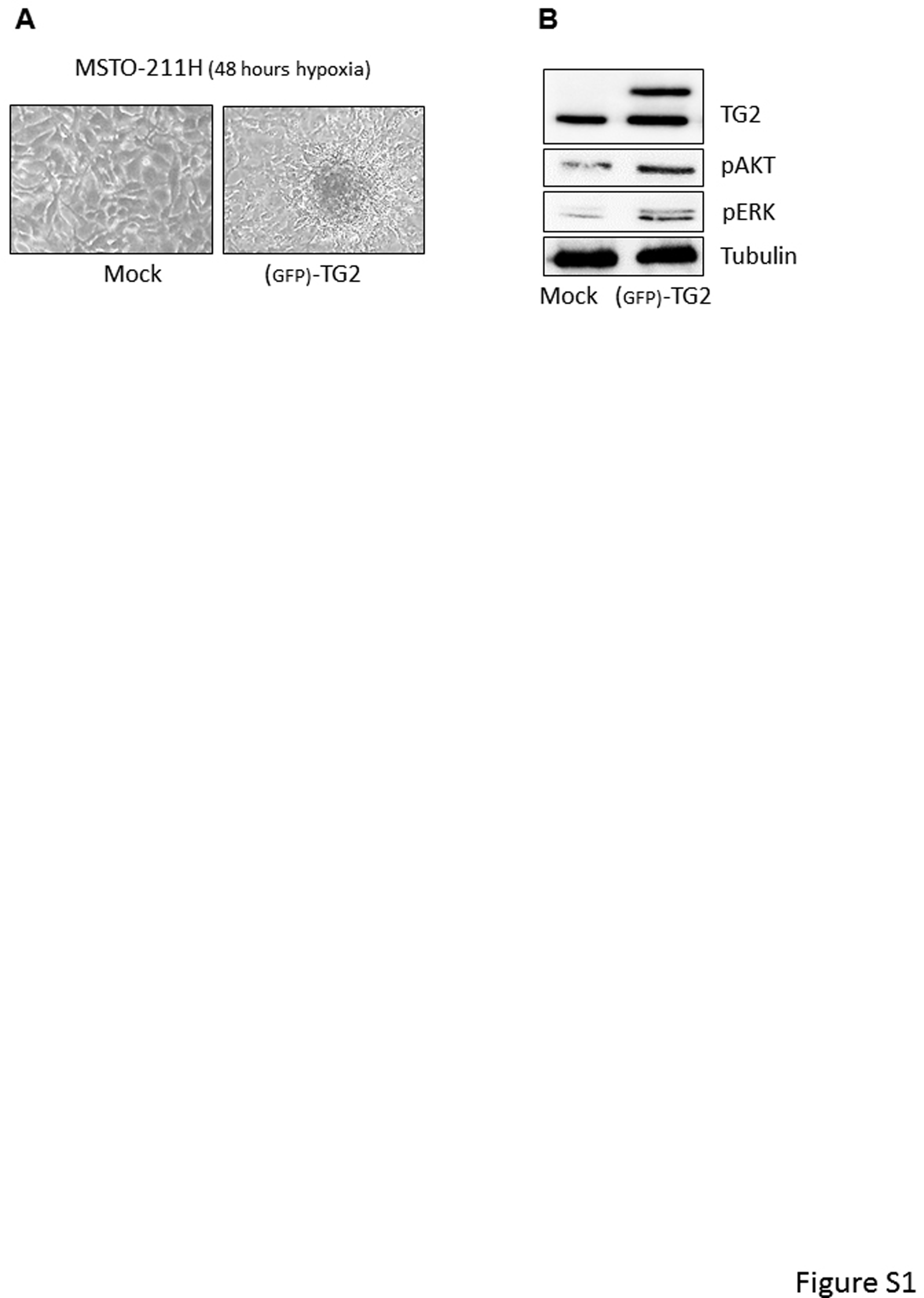

Supplement: Supplementary Figure S1 [file cddis201730x1.tif]

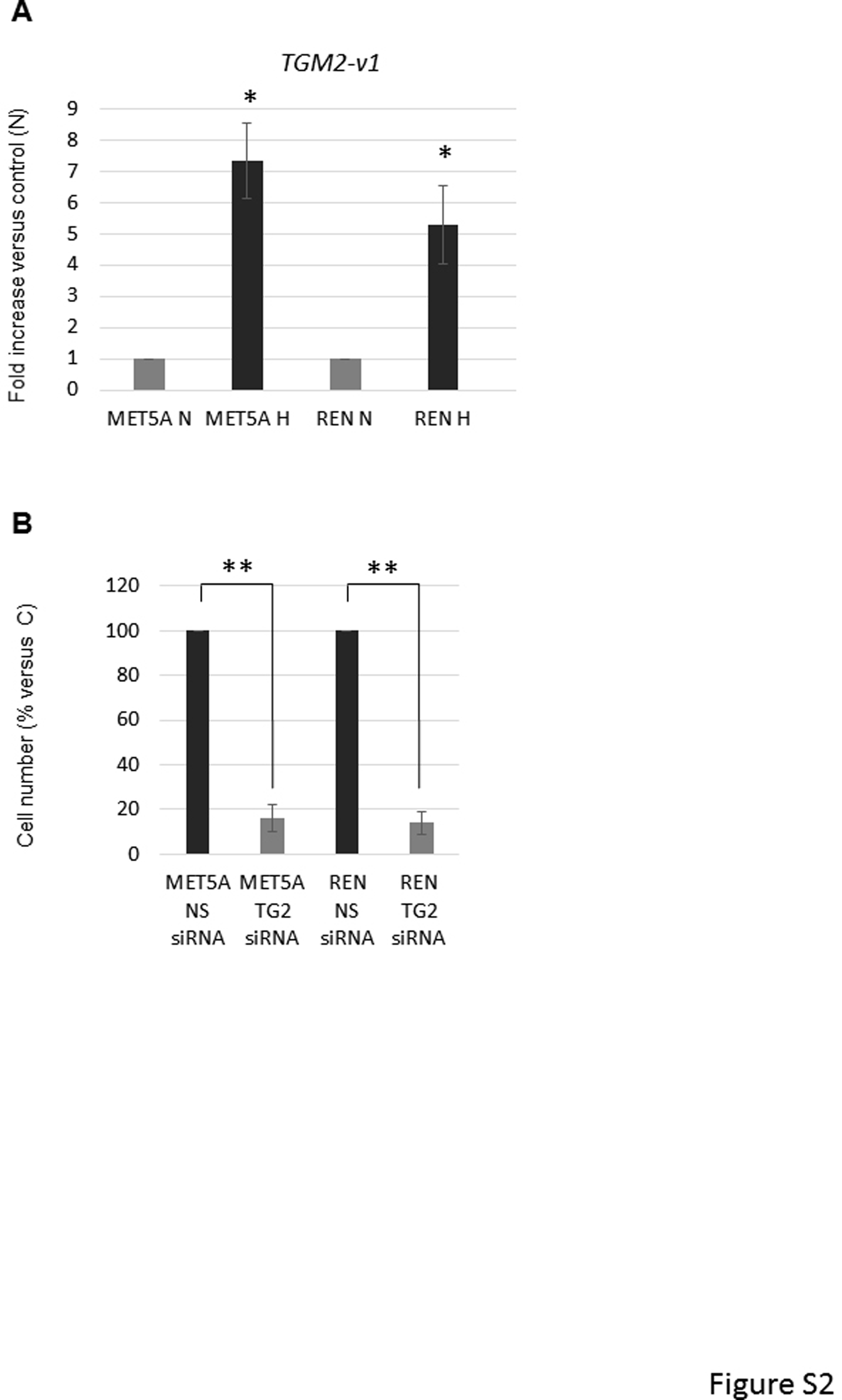

Supplement: Supplementary Figure S2 [file cddis201730x2.tif]

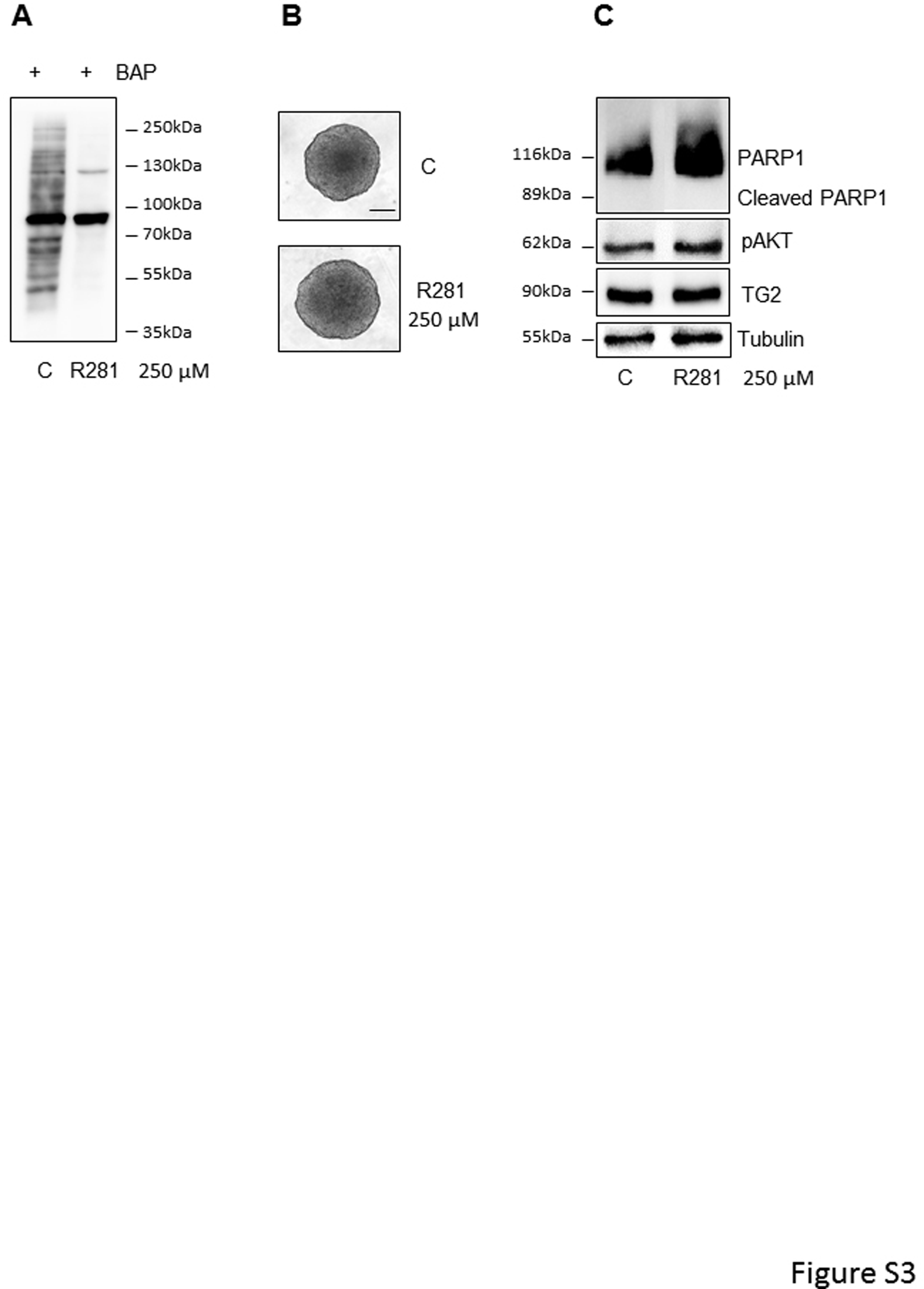

Supplement: Supplementary Figure S3 [file cddis201730x3.tif]
